# Opposite Cannabis-Cognition Associations in Psychotic Patients Depending on Family History

**DOI:** 10.1371/journal.pone.0160949

**Published:** 2016-08-11

**Authors:** Ana González-Pinto, Itxaso González-Ortega, Susana Alberich, Sonia Ruiz de Azúa, Miguel Bernardo, Miquel Bioque, Bibiana Cabrera, Iluminada Corripio, Celso Arango, Antonio Lobo, Ana M. Sánchez-Torres, Manuel J. Cuesta, the PEPs Group

**Affiliations:** 1 Centre for Biomedical Research in the Mental Health Network (CIBERSAM), Madrid, Spain; 2 Department of Psychiatry, Araba University Hospital, University of the Basque Country, Vitoria, Spain; 3 Barcelona Clinic Schizophrenia Unit, Neuroscience Institute, Hospital Clinic of Barcelona, Barcelona, Spain; 4 Department of Psychiatry and Clinical Psychobiology, University of Barcelona. Institut d'Investigacions Biomèdiques August Pi i Sunyer (IDIBAPS), Barcelona, Spain; 5 Department of Psychiatry, Institut d'Investigació Biomèdica-Sant Pau (IIB-SANT PAU), Hospital de la Santa Creu i Sant Pau, Universitat Autònoma de Barcelona (UAB), Barcelona, Spain; 6 Child and Adolescent Psychiatry Department. Gregorio Marañón General University Hospital. School of Medicine, Universidad Complutense, IiSGM, Madrid, Spain; 7 University of Zaragoza, Zaragoza, Spain; 8 Aragon Institute for Health Sciences (IIS Aragón), Zaragoza, Spain; 9 Department of Psychiatry, Navarre Hospital Complex, Pamplona, Spain. IdiSNA, Navarre Institute for Health Research, Pamplona, Spain; Maastricht University, NETHERLANDS

## Abstract

The objective of this study is to investigate cognitive performance in a first-episode psychosis sample, when stratifying the interaction by cannabis use and familial or non-familial psychosis. Hierarchical-regression models were used to analyse this association in a sample of 268 first-episode psychosis patients and 237 controls. We found that cannabis use was associated with worse working memory, regardless of family history. However, cannabis use was clearly associated with worse cognitive performance in patients with no family history of psychosis, in cognitive domains including verbal memory, executive function and global cognitive index, whereas cannabis users with a family history of psychosis performed better in these domains. The main finding of the study is that there is an interaction between cannabis use and a family history of psychosis in the areas of verbal memory, executive function and global cognition: that is, cannabis use is associated with a better performance in patients with a family history of psychosis and a worse performance in those with no family history of psychosis. In order to confirm this hypothesis, future research should explore the actual expression of the endocannabinoid system in patients with and without a family history of psychosis.

## Introduction

Cannabis is the most commonly used illicit drug in psychosis [1]. Moreover, it is a controversial drug, with detractors and supporters, which has a high social impact. There is strong evidence that long-term cannabis use is associated with decreased functionality in psychosis [2]. However, recent meta-analyses have also found associations between a history of cannabis use and improved cognition in psychosis [3,4]. Furthermore, the study by Yücel et al. [4] found that patients with first-episode psychosis (FEP) and earlier cannabis use had better cognitive performance than patients with later onset of use. There are also numerous contrasting findings in relation to neurobiological research on cannabis and cognition in psychosis. Whereas some studies have revealed cognitive impairment [5–10] and deficits in grey matter, lateral ventricle enlargement, and brain abnormalities [11–13] in FEP cannabis users, other authors have suggested that FEP related to cannabis use is associated with better cognitive functioning and fewer brain abnormalities [4,14–16]. In addition to these controversial results, a deleterious effect of cannabis on cognition has been demonstrated in both animal models [17] and healthy subjects [18]. However, an increased vulnerability to the deleterious effect of cannabis on cognition has been found in patients with schizophrenia as compared to healthy controls, specifically in the memory and learning domains [9]. In contrast, other authors have reported that FEP patients who used cannabis have lower cognitive impairment relative to healthy controls in relation to visual memory, working memory, planning and reasoning domains [6]. Moreover, shared effects of genetics and family environment have been suggested as influential in the development of brain structure and cannabis use. Pagliaccio et al. [6] have reported that cannabis exposure is associated with small subcortical structures in a large sample of normal twins/siblings discordant for cannabis abuse. However, not only were these variations within the normal range, they were also primarily attributed to familial factors, rather than to a direct neurotoxic effect [6]. In respect of cognitive impairment, psychotic patients with a family history (FH) of psychosis had previously shown worse cognition than patients with no FH of psychosis [19–24].

As the data regarding the relation between cannabis use and cognition are highly controversial, and cognitive deficits are core symptoms of schizophrenia, it was considered possible that not all cases of psychosis had the same cognitive associations with cannabis use. Therefore, we focused on FH of psychosis, and split the sample into two different groups according to this variable: people with FEP and with or without FH of psychosis.

We conducted a study to investigate the associations between cannabis use and cognition in patients with psychosis, distinguishing the interactions by having a familial vs. no familial history of psychosis. The specific aims of the study were: 1) to compare cognition between patients with a family history of psychosis (FH^+^) and patients with no family history of psychosis (FH^-^) as regards cannabis use; 2) to determine the independent effect of cannabis use and the FH of psychosis on cognition and the interaction between these two factors; and 3) to analyse the associations between cannabis use and cognition in healthy controls and to compare them with those in FEP patients.

## Materials and Methods

### Subjects

This work was part of the “Phenotype-genotype and environmental interaction: application of a predictive model in first psychotic episodes” study (PEPs study, from its acronym in Spanish) [25]. Patients were matched with healthy controls by age (±10%), sex and parental socio-economic level, and assessed using the Hollingshead-Redlich index of social position [26] (±1 level).

Patients and controls were required to be fluent in Spanish and to provide informed consent. The clinicians who contributed to recruitment of the sample assessed the capacity of patients to provide informed consent via a clinical interview. In the cases of patients not able to provide consent, or of patients who were minors, a legally authorized representative gave consent on behalf of the participants. Moreover, patients were required to have had psychotic symptoms of less than 12 months’ duration.

Exclusion criteria for patients and controls were: mental retardation according to the DSM-IV [27] (including both an IQ below 70, as determined using the test described in Table 1, and poor functionality), a history of head trauma with loss of consciousness, and organic illness with mental repercussions. Controls were also excluded if they had suffered, or were suffering, from psychotic disease, were experiencing major depression according to the DSM-IV [28,29], or had a first-degree family member with a history of psychotic disease.

**Table 1:** Development of the cognitive domains.

Cognitive domain	Neuropsychological subtests	Description of test used for cognitive domain scores
Estimated premorbid IQ	Vocabulary subtest of WAIS-III^a^/ WISC-IV^b^	Give oral definitions for words. Measure: direct score and standardized score.Estimated premorbid IQ is calculated from the standardized score: (SS × 5) + 50.
Processing speed	Trail Making Test^c^-form A	Connect in proper order, by making pencil lines, 25 encircled numbers randomly arranged on a page.Measure: time to complete this (form A)
	Stroop Test word and colour condition^d^	Read the words and the colours of a series of XXXX as quickly as possible in 45 s. Measure: number of items completed.
Attention	CPT-II^e^	Respond to a series of letters on a computer screen by pressing a key when you detect letters other than the letter “X”. The assessment contains six blocks that vary in the rate of submission of the letters. Measure: mean response sensitivity (D-prime).
Verbal memory	TAVEC^f^	Recall as many words as possible from a list of 16 words read aloud by the tester. The procedure is repeated five times, and recall is tested immediately and after a delay. Measure: total number of words recalled after the five trials, immediately and delayed.
Working memory	Digit Span subtest of the WAIS-III^a^/ WISC-IV^b^	Repeat a number sequence in the same and order as presented and in reverse. Measure: total number of series correctly repeated forwards, backwards, and the sum of both.
	Letter and number sequencing of the WAIS-III^a^/ WISC-IV^b^	Listen to a combination of numbers and letters read aloud by the tester and reorganize the sequence, listing first the numbers in ascending order and then the letters in alphabetical order. Measure: number of correct sequences.
Executive function	FAS test^g^	Generate as many words as possible beginning with F, A and S, in three separate trials of 60 s. Measure: the sum of all correct responses
	Animal words^h^	Produce as many animal names as possible in a 1-min period. Measure: number of correct responses.
	Trail Making Test^c^-form B	Draw lines connecting characters, alternating sequentially between numbers and letters. Measure: time to complete this (form B), B/A ratio (time to complete form B divided by time to complete form A).
	WCST^i^	Complete a complex task of categorization set shifting, and respond to feedback from the computer.Measure: number of completed categories and percentage of conceptual responses, total errors and perseverative errors.
	Stroop Test^d^- interference condition	Name the colour in which the colour names are printed, disregarding their verbal content. Measure: Interference Index (WC − WxC /W+ C).
Cognitive Global Index	Processing speed	A Global Cognitive Index score was calculated by averaging scores for all cognitive domains, except the estimated premorbid IQ.
	Attention	
	Verbal memory	
	Working memory	
	Executive function	

^a^Wechsler Adult Intelligence Scale III (WAIS-III) [33].

^b^Wechsler intelligence Scale for children-IV (WISC-IV) [34].

^c^Trail Making Test [35].

^d^Stroop Test [36].

^e^Conners’ Continuous Performance Test (CPT-II) [37].

^f^Test de Aprendizaje Verbal España-Complutense (TAVEC) [38].

^g^Controlled Oral Word Association Test (FAS test) [39].

^h^Animal words [40].

^i^Wisconsin Card Sorting Test (WCST) [41].

The study was approved by the following clinical research ethics committees: Araba University Hospital, Hospital Clinic of Barcelona, Gregorio Marañon General University Hospital, Navarre Hospital Complex, and the centres included in the PEPs Group.

Initially, 335 FEP patients and 253 healthy controls were included in the study. Of these subjects, 69 (53 patients and 16 controls) were excluded for not having completed seven or more neuropsychological tests. Moreover, 14 patients were excluded because of a lack of data on FH of psychosis (first- or second-degree). As a result, the final study sample consisted of 268 patients and 237 controls. Patients were distributed into two groups, depending on whether or not they had an FH of psychosis. A total of 88 patients were FH^+^, whereas 180 were FH^-^. For cognitive comparison, each group was split into two subgroups: one group of cannabis users (FH^-^ = 73; FH^+^ = 34; controls = 37) and one group of non-users (FH^-^ = 107; FH^+^ = 54; controls = 200).

### Data collection

Socio-demographic and clinical data were collected at baseline and cognitive assessment was performed at two months follow-up, in order to ensure the psychopathologic stability of patients. The assessment protocol is fully discussed in the paper by Bernardo et al. [25].

The socio-demographic variables included were sex, age, civil (marital) status, educational background, parental socio-economic status, and occupation.

Adult patients were diagnosed using the SCID-I and II [28], whereas the K-SADS-PL [29] was used for patients aged below 18 years, in accordance with the DSM-IV criteria. Patients were classified into three groups according to diagnosis: 1) those with schizophrenia-spectrum disorders, including schizophrenia, schizoaffective disorders and schizophreniform disorders; 2) those with affective-spectrum disorders, such as bipolar disorder, manic and depressive episodes; and 3) those with other psychoses, such as substance-induced psychosis, brief psychotic disorders, and psychoses not otherwise specified.

Pharmacological treatment was also recollected at baseline. Antipsychotic daily doses were converted to chlorpromazine equivalents [30].

Use of cannabis and other substances (including alcohol and tobacco) was assessed in patients and healthy controls using the European Adaptation of a Multidimensional Assessment Instrument for Drug and Alcohol Dependence (EuropASI), and was categorized into four groups (*no use*, *use*, *abuse* and *dependence*) according to EuropASI scores [31]. Age of first use was also recorded.

The Family History of Psychosis-Related Disorder (FHPRD) was assessed using a protocol that evaluated first- and second-degree family history of mental illnesses, according to DSM-IV criteria. In the case of patients under 18 years of age, at least one adult relative was interviewed for each participant. The FHPRD was positive when one or several members of the family (first- or second-degree) presented a diagnosis of psychotic disorder with delusions or hallucinations requiring psychiatric treatment.

Cognitive performance was evaluated using the following cognitive domains: processing speed, attention, verbal memory, working memory, executive function and estimated premorbid IQ. A Cognitive Global Index (CGI) was calculated by averaging the scores of the cognitive domains, except the estimated premorbid IQ, which was used as an adjustment variable in the statistical analyses. Development of these domains is reported in the papers by Bernardo et al. [25] and Cuesta et al. [32] (Table 1).

### Statistical Analyses

Differences in socio-demographic variables between FH^+^ and FH^-^ patients and healthy controls were analysed using one-way ANOVA tests for continuous variables and the Chi-Squared test for categorical variables. For those significant differences between groups, a post-hoc analysis was performed.

With regard to cognitive measures, Z-scores were derived, based on the means and standard deviations of the control group to standardize the different measurement scales used in the neuropsychological tests. Composite scores for cognitive domains represented by more than one measure were calculated by taking the mean of all the Z-scores included in each cognitive domain. Cronbach’s alpha was calculated to assess the internal consistency of the composite scores, and gave a value of 0.84 for the processing speed domain, 0.82 for verbal memory, 0.73 for working memory and 0.76 for executive function. Since all coefficients indicate moderate reliability, we decided to use these domains to analyse the relationship between cannabis use and cognition.

One-way ANOVA tests were performed to assess differences in patient cognition between groups of cannabis users (no use, use, abuse and dependence) with/without FH of psychosis. Subsequently, Bonferroni post-hoc tests were used to determine the groups for which differences in ANOVA tests were found. Differences between controls with/without cannabis use at baseline were compared using Student’s *t*-test for independent samples.

Hierarchical-regression models were developed to assess the association between FH of psychosis, cannabis use and cognition. Cognitive domains were taken as dependent variables in these models. Cannabis use at baseline (presence/absence) and the subject variable (FH^+^ patient, FH^-^ patient, controls) were introduced as independent variables. In addition, the interaction between cannabis use and subject variable was also considered in order to assess the combined effect of these variables on cognition. We identified the following variables as potential confounders: sex, age, occupation, educational level, civil status, alcohol and tobacco consumption, and IQ, and, after verifying the independence of significant potential confounders (occupation, educational level, alcohol, tobacco consumption and estimated premorbid IQ), the models were finally adjusted for estimated premorbid IQ and alcohol consumption.

To facilitate interpretation and comparison of groups in the hierarchical-regression models developed to analyse the cognitive domains, FH^-^ patients were used as the reference group, while, for cannabis use at baseline, non-user patients was the reference category.

All statistical analyses were performed using the R statistical package, version 3.1.2 [42].

## Results

### Baseline characteristics

The baseline characteristics for controls and FH^+^ and FH^-^ patients are summarized in Table 2. Patients (FH^+^ and FH^-^) were less likely to have a university education (*P*<0.001) and were more likely to be tobacco (P<0.001) and cannabis users than were controls (*P*<0.001). In contrast, controls were more commonly active workers (46% vs. 19.5% and 12.8%, respectively, *P*<0.001) and alcohol users (65% vs. 48.2% and 47.9%, respectively, *P*<0.001). Statistically significant differences were also found between controls and patients in respect of estimated premorbid IQ (*P<*0.001), with FH^+^ patients having the lowest scores and close to 16 points below that for controls. In the post-hoc analysis, all the significant differences were between patients (FH^+^ and FH^-^) and controls. Moreover, a comparison of estimated premorbid IQ between cannabis users and non-users showed that the latter group had a significantly higher IQ than patients who were cannabis users. On analysis, this relationship was only found to be significant in the FH^-^ group. Finally, antipsychotic doses for both groups were compared, and there were no significant differences.

**Table 2:** Baseline characteristics of controls and FH^+^ and FH^-^ patients.

		FH^+^ patients (n = 88)	FH^-^ patients (n = 180)	Controls (n = 237)	Statistic
Sex	Female	30 (34.1%)	54 (30%)	84 (35.4%)	X^2^ = 1.75, *P* = 0.417
Age		24.17 (5.75)	23.47 (5.80)	24.33 (6.43)	F = 1.83, *P* = 0.161
Educational level	Primary education	19 (21.6%)	41 (22.8%)	17 (7.2%)	**X**^2^ **= 60.74, *P*<0.001**
	Secondary education	57 (64.8%)	112 (62.2%)	119 (50.2%)	
	University	12 (13.6%)	27 (15%)	101 (42.6%)	
Civil status	Single	73 (83%)	162 (90%)	200 (84.4%)	X^2^ = 2.48, *P* = 0.242
	Married	6 (6.9%)	11 (6.1%)	19 (8.2%)	
	Other	9 (10.2%)	7 (3.9%)	18 (7.6%)	
Occupation	Working	17 (19.5%)	23 (12.8%)	109 (46%)	**X**^2^ **= 94.85, *P*<0.001**
	Student	39 (44.8%)	80 (44.4%)	109 (46%)	
	Other	31 (35.6%)	77 (42.8%)	19 (8%)	
Current alcohol use	Yes	39 (48.2%)	81 (47.9%)	145 (65%)	**X**^2^ **= 13.91, *P* = 0.001**
Current tobacco use	Yes	58 (68.2%)	108 (63.5%)	77 (34.8%)	**X**^2^ **= 43.64, *P*<0.001**
Current cannabis use	Yes	34 (38.6%)	73 (43.2%)	37 (16.6%)	**X**^2^ **= 37.25, *P*<0.001**
Estimated premorbid IQ		91.53 (16.56)	92.86 (14.64)	107.48 (14.18)	**F = 66.37, *P*<0.001**
Antipsychotic dose (mg/day)		592.84 (476.27)	550.26 (431.72)	NA	t = -0.73, p = 0.467

### Neuropsychological results

S1 Table shows the Z-scores for the cognitive domains for each group, depending on cannabis use and FH of psychosis.

**Fig 1:**
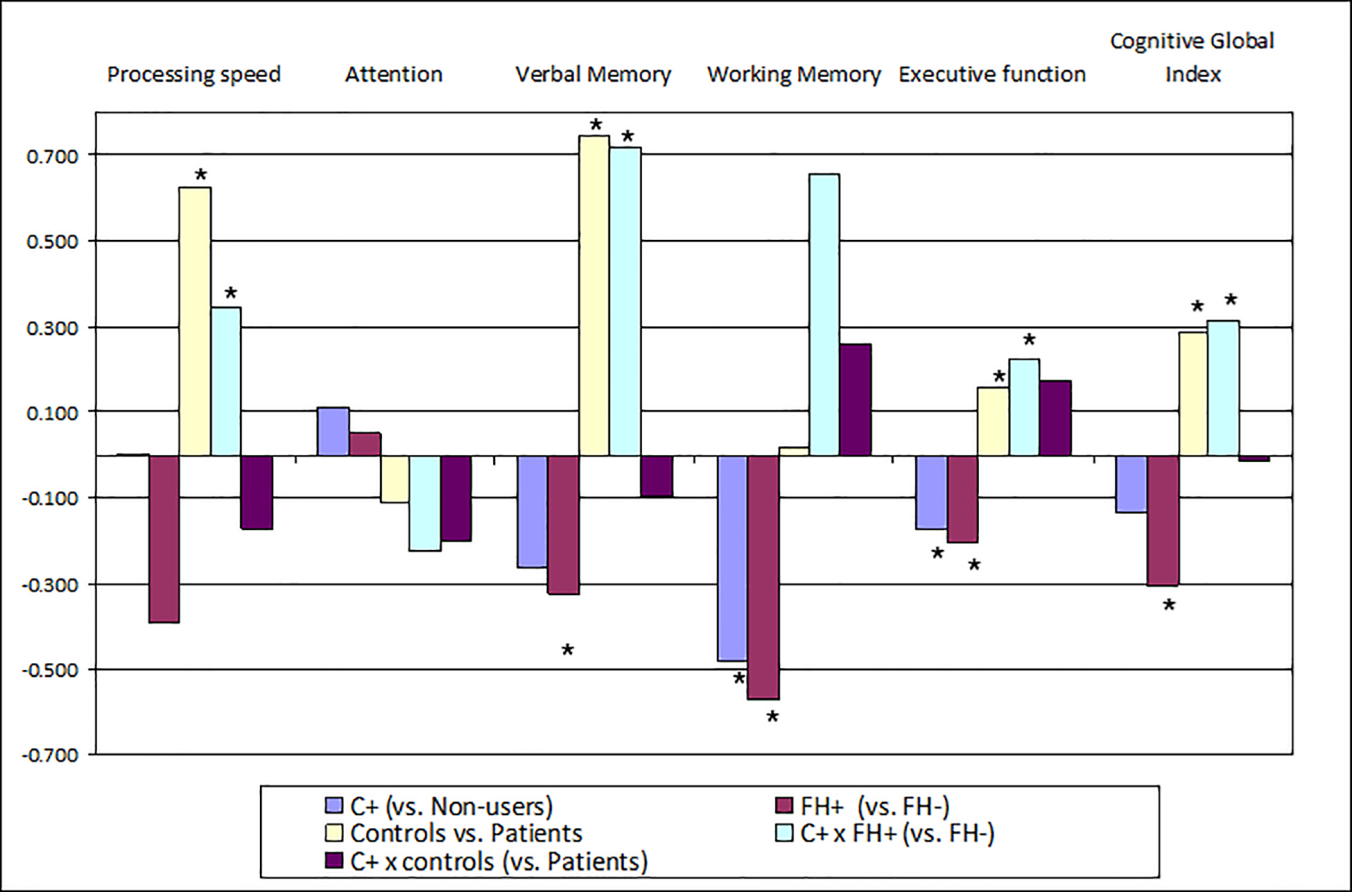
Graphical representation of the regression models.

**Fig 2:**
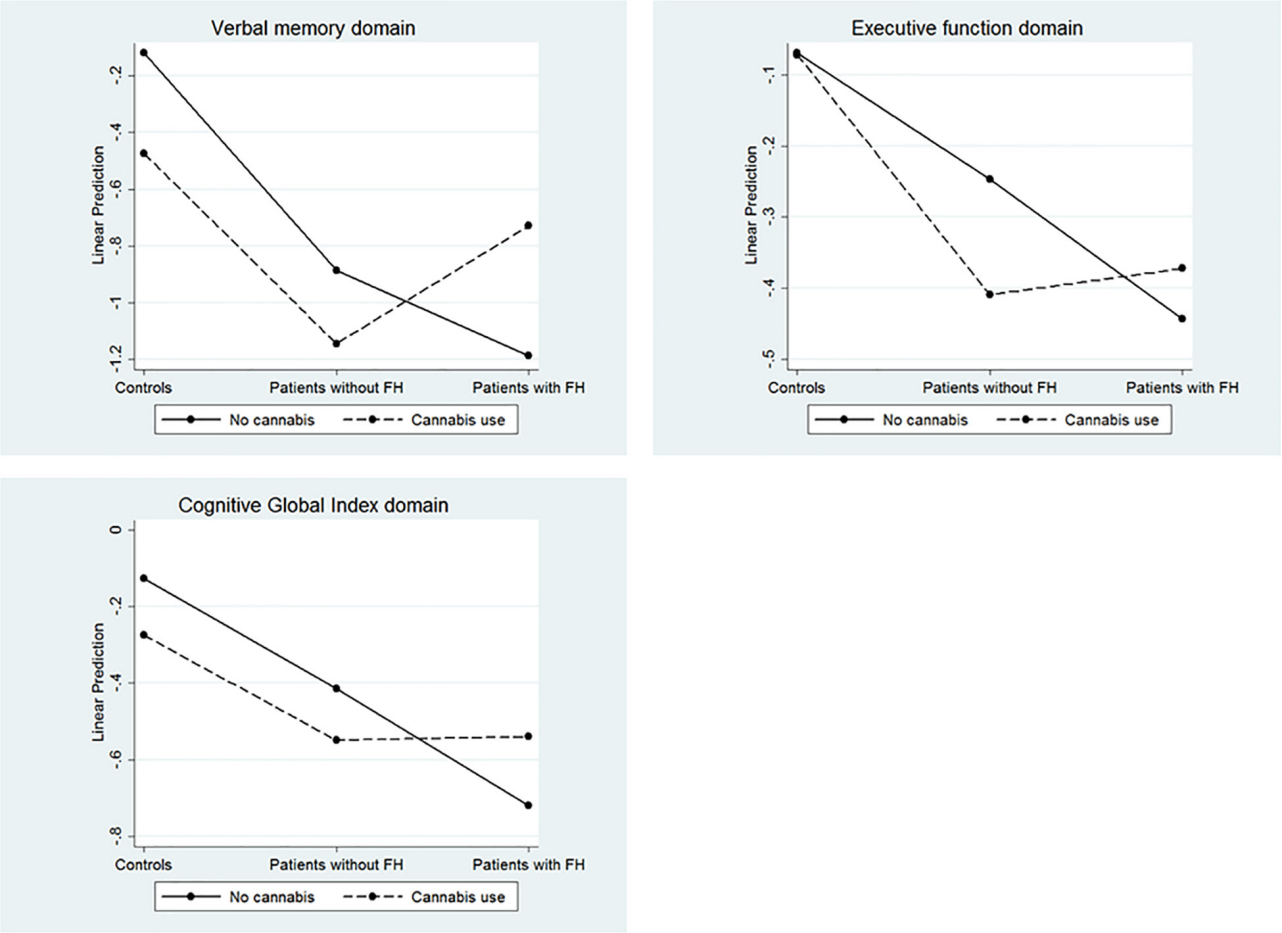
Statistically significant interactions between cannabis use and subject type.

Statistically significant associations were found in all cognitive domains, except for Attention in regression models. The results are summarized in Table 3 and Fig 1. Fig 2 shows the interactions between subjects and cannabis use in the domains in which a statistically significant interaction was found.

**Table 3:** Hierarchical-regression models between subject type, cannabis use and cognition.

	Variables in the model^a^	β	*P*-value	95% CI	R^2^
Processing speed	Cannabis: users (vs. ref)	0.002	0.990	-0.297, 0.280	0.315
	Subject type: FH^+^ (vs. ref)	-0.392	**0.012**	-0.680, -0.071	
	Subject type: Controls (vs. ref)	0.626	**0.003**	0.410, 0.885	
	Cannabis*Subject type: users*FH^+^ patients (vs. ref)	0.344	0.170	-0.139, 0.844	
	Cannabis*Subject type: users*controls (vs. ref)	-0.174	0.417	-0.590, 0.248	
Attention	Cannabis: users (vs. ref)	0.113	0.253	-0.073, 0.308	0.055
	Subject type: FH^+^ (vs. ref)	0.053	0.604	-0.152, 0.247	
	Subject type: Controls (vs. ref)	-0.113	0.160	-0.280, 0.033	
	Cannabis*Subject type: users*FH^+^ patients (vs. ref)	-0.224	0.187	-0.556, 0.103	
	Cannabis*Subject type: users*controls (vs. ref)	-0.199	0.166	-0.476, 0.082	
Verbal memory	Cannabis: users (vs. ref)	-0.262	0.079	-0.550, 0.036	0.378
	Subject type: FH^+^ (vs. ref)	-0.324	**0.046**	-0.603, 0.004	
	Subject type: Controls (vs. ref)	0.748	**<0.001**	0.530, 1.007	
	Cannabis*Subject type: users*FH^+^ patients (vs. ref)	0.720	**0.004**	0.219, 1.211	
	Cannabis*Subject type: users*controls (vs. ref)	-0.096	0.659	-0.528, 0.333	
Working memory	Cannabis: users (vs. ref)	-0.479	**0.043**	-0.936, -0.019	0.116
	Subject type: FH^+^ (vs. ref)	-0.571	**0.020**	-1.021, -0.066	
	Subject type: Controls (vs. ref)	0.018	0.927	-0.325, 0.423	
	Cannabis*subject type: users*FH^+^ patients (vs. ref)	0.654	0.101	-0.129, 1.427	
	Cannabis*subject type: users*controls (vs. ref)	0.260	0.447	-0.417, 0.916	
Executive Function	Cannabis: users (vs. ref)	-0.171	**0.019**	-0.305, -0.018	0.266
	Subject type: FH^+^ (vs. ref)	-0.208	**0.005**	-0.342, -0.051	
	Subject type: controls (vs. ref)	0.159	**0.007**	0.062, 0.294	
	Cannabis*subject type: users*FH^+^ patients (vs. ref)	0.224	**0.047**	0.007, 0.476	
	Cannabis*subject type: users*controls (vs. ref)	0.175	0.091	-0.045, 0.363	
Cognitive global Index	Cannabis: users (vs. ref)	-0.134	0.118	-0.303, 0.034	0.407
	Subject type: FH^+^ (vs. ref)	-0.305	**<0.001**	-0.475, -0.134	
	Subject type: controls (vs. ref)	0.287	**<0.001**	0.154, 0.420	
	Cannabis*subject type: users*FH^+^ patients (vs. ref)	0.314	**0.031**	0.029, 0.598	
	Cannabis*subject type: users*controls (vs. ref)	-0.014	0.910	-0.252, 0.225	

^a^ Variables in the model: IQ, alcohol at baseline, cannabis use at baseline (yes/no), subject type (FH^-^ patients/ FH^+^ patients/controls).

References groups: for the use of cannabis at baseline: no use; for the subject type variable: FH^-^ patients.

Controls had better scores than FH- patients for the processing-speed domain, while FH+ patients were the worst group, with the lowest scores. Cannabis use had no impact on any group.

A significant interaction between cannabis use and the subject-type variable was obtained for the verbal memory domain. As a consequence, whereas controls attained the best values in this domain, the group of FH^+^ cannabis users had the best estimated score amongst patients, while FH^+^ non cannabis users had the worst score (Fig 2).

**Fig 3:**
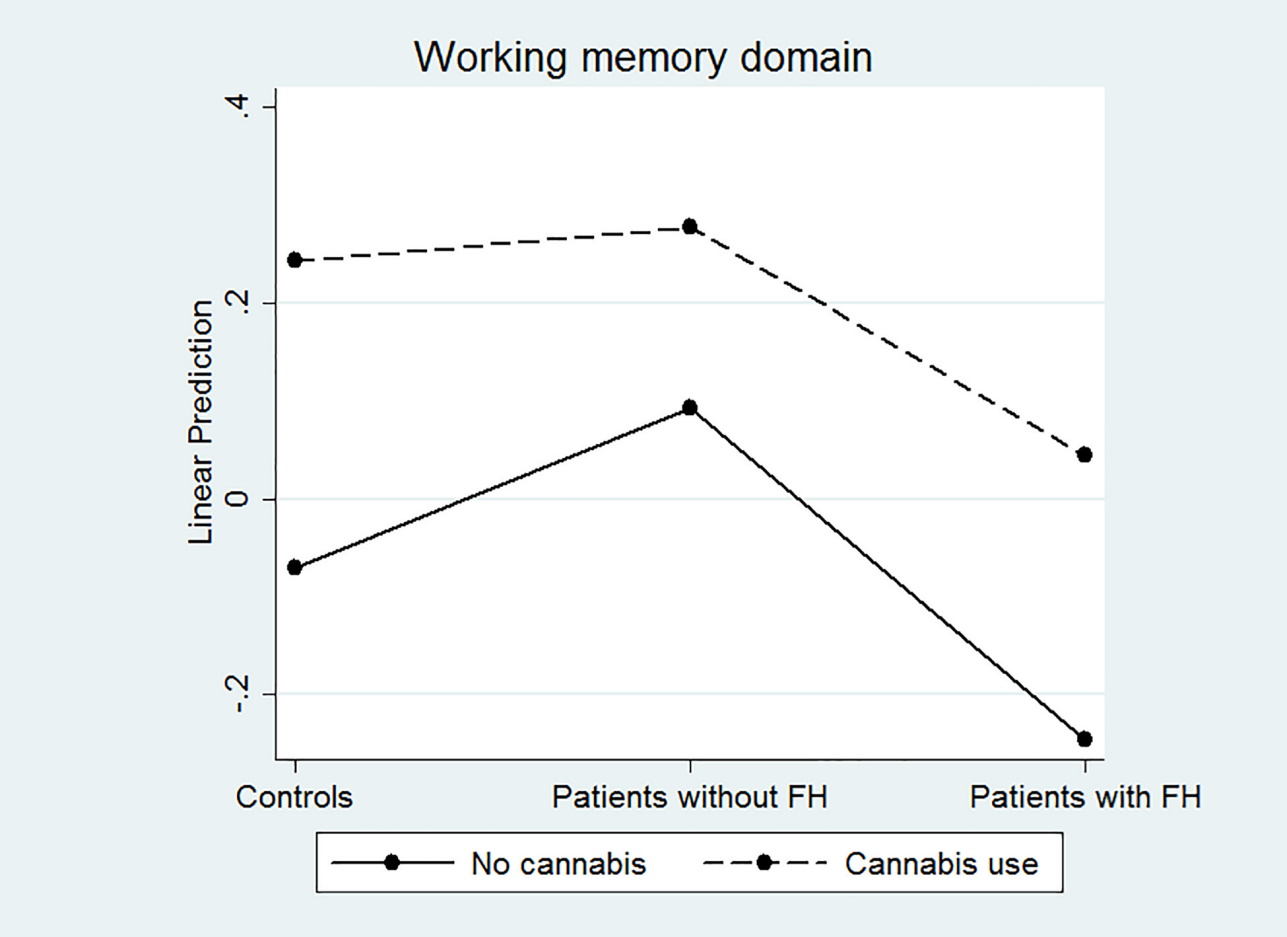
Association between cannabis use and working memory.

Both cannabis use and FH were significantly related to inferior working memory, although their interaction was not significantly associated with this domain. As a consequence, cannabis users (regardless of whether they were patients or controls) had lower scores in this domain than did non-users. The same occurred with FH^+^ patients, who had worse scores than FH^-^ ones (regardless of whether or not cannabis was used) (Fig 3).

With regard to executive function, a statistically significant interaction with cannabis use was obtained in FH^+^/FH^-^ patients (Table 3). The group with the best estimated scores in this domain was that of FH^-^ non-cannabis users, whereas FH^+^ non-user patients had the worst estimated executive function. The control group had the highest scores, irrespective of cannabis consumption (Fig 2).

Finally, both cannabis use and the subject-type variable interacted significantly in the Global Cognitive Index (GCI) domain (Table 3). Indeed, as was the case with the executive-function domain, FH^-^ patients who did not use cannabis were the patient group with the best estimated score in the GCI, while FH^+^ non-cannabis users had the worst estimated score in this domain. Controls (using cannabis or not) gave the highest scores in the GCI (Fig 2).

## Discussion

The two main findings of this study are that cannabis use is associated with inferior working memory in FEP patients, regardless of having a positive FH for a psychosis-related disorder, and that cannabis is clearly associated with inferior cognitive performance in FH^-^ patients in relation to the verbal-memory, executive-function, and GCI domains, while FH^+^ cannabis users showed better cognition in these domains. These findings are relevant and have translational value, because treatment of cannabis use requires different approaches, depending on FH of psychosis. The association found between cannabis use and cognitive deficits in these patients varied depending on FH. These results clarify some of the controversies concerning the association between cannabis use and cognitive deficits, and should be taken into consideration for the prevention and treatment of these patients.

As shown in Table 3, cannabis users (patients and controls) had worse working memory and executive function to non-users, when FH was not considered. Acute administration of THC causes more deleterious cognitive effects in patients with psychosis than in healthy control subjects [9]. Some studies have found cognitive impairment in FEP cannabis-user patients compared with control subjects [9] and cognitive deficits in processing speed and verbal memory [43], along with brain abnormalities [11–13]. In contrast, other authors have reported that psychosis patients who use cannabis have better cognitive functioning in some domains than do non-cannabis users [4,14–16], with fewer brain abnormalities and less attention and executive impairment than patients who have never used cannabis [44].

These conflicting findings are probably mediated by different moderators. Yücel et al. [4] considered that there is probably a subgroup of cannabis users with psychosis who have better premorbid adjustment and cognitive performance. Meijer et al. [43] also found that current cannabis use is associated with poorer cognitive performance, but that lifetime cannabis users may have a higher cognitive potential. This does not mean that cannabis has a beneficial effect on cognition; on the contrary, it worsens cognitive performance, although some of these individuals had better premorbid capacities. Our study was performed on FEP, and all patients were included at the same time, consequently limiting the possible moderating effect of time.

According to Van Winkel et al. [10], the deleterious effect of cannabis use on cognition in patients with psychosis may be moderated by the *AKT1* gene. These authors showed that cannabis-user patients who carried the CC variant in the *AKT1* gene performed worse in attention tests than patients with this genotype who did not use cannabis, whereas cannabis users with the TT variant in *AKT1* performed as well as, or better than, cannabis non-users [45]. We have not attempted to identify genetic differences amongst our patients in this study.

The different interaction of cannabis use depending on the FH of psychosis has not been demonstrated previously. Although we cannot state causal relationships, this interaction is sufficiently large as to be unlikely to be due to chance. This suggests that the FH-cannabis interaction affects more complex cognitive functions (for example, executive function), which depend on multiple cognitive processes, more than it does basic functions (for example, attention). These data support previous studies on cognition and cannabis use in patients with schizophrenia [7,8]. However, FH^+^ cannabis users show better functioning in complex cognitive domains, as reported in recent meta-analyses [4]. This finding must be interpreted with caution because, considering the data as a whole, cannabis use is associated with worse working memory regardless of having FH of psychosis or not. Our findings do not support the idea of better cognitive performance in patients with cannabis use; rather, they suggest an FH of psychosis as a possible factor contributing to the controversial results found in the literature.

A possible explanation for the differential effects of cannabis depending on FH in patients could be a distinct effect of cannabis compounds on the central nervous system of FH^+^ patients. Although the endocannabinoid system is involved in the development of cognitive deficits associated with cannabis use and schizophrenia [46], it is well known that cannabidiol (CBD) and tetrahydrocannabinol (THC) have opposing effects in various brain structures involved in psychiatric disorders, notably the striatum, cingulate, prefrontal cortex, hippocampus and amygdala. CBD might protect neurons against the possible neurotoxic effects of THC, meaning that it could inhibit the cognitive and psychotomimetic effects of THC. Moreover, CBD has been found to protect users against both psychotic symptoms and memory impairment [47]. One possible explanation for our findings is that the protective role of CBD is expressed better in FH^+^ patients than in FH^-^ ones. Future research could explore the real expression of the endocannabinoid system in FH^+^ and FH^-^ patients to confirm this hypothesis. Our findings suggest that FH is an important factor to consider when studying the effects of cannabis on cognition. Our results support some differences in cognitive deficits when taking into account cannabis use and FH. Cannabis and FH are separately associated with poorer cognitive functioning, while their interaction has no significant effects on cognition and impacts differently depending on whether FH is present or not.

When comparing cognition without considering cannabis use, FH^+^ patients had worse CGI, processing speed, verbal and working memory, and executive function than FH^-^ patients. Previous studies have shown that FH^+^ patients have greater cognitive deficits [19–24]. Moreover, some research has shown that FH^+^ patients and patients with a high clinical risk of psychosis have cognitive deficits, and that co-occurring attenuated symptoms and genetic risk are related to more severe cognitive deficits [20,48]. Bora et al. [20] suggested that cognitive impairment might predate the onset of FEP and could be genetically transmitted in some patients. In fact, there is evidence of cognitive impairment in relatives of patients with psychosis [49–55]. In a study performed by Gur et al. [53], unaffected offspring, monozygotic and dizygotic co-twins, and other relatives, had deficits in episodic memory, working memory, and attention. Cognitive deficits have also been found in siblings when compared to healthy control subjects [56,57] and in the offspring of patients with schizophrenia [23,53]. Other studies that have compared IQ in patients with and without FH have also found that both patients and their relatives had lower global IQ than healthy controls [19,21–23], while patients with no affected relatives had higher estimated premorbid IQ [58]. A recent study conducted by Derks et al. [59] concluded that FH^+^ patients showed more positive symptoms than FH^-^ patients, but no differences were found as regards IQ, cannabis abuse, or age at onset. In our sample of FEP patients, patients with and without FH also had a poorer estimated premorbid IQ than healthy controls; there were no differences between patient groups.

This study has several strengths. First, it is a multi-centre study that includes a large sample of patients and controls recruited in multiple Spanish psychiatric admission centres for acute psychosis, meaning that generalizability of the results is ensured. Second, the study comprised child and adolescent patients, in addition to adults. This wide age window allows the collection of a more representative sample, with an average age (23.63±5.9) lower than that for other studies with large FEP cohorts (OPUS trial: 26.6±6.4; EUFEST trial: 26±5.6), which did not include child and adolescent patients [60,61]. Third, unlike other FEP studies, such as EUFEST [62,63], the neuropsychological battery used was extensive and covered the areas proposed by the NIMH-MATRICS consensus (except visual memory) [64,65].

Nevertheless, this study has some limitations that must be taken into account when interpreting the findings. These limitations include the naturalistic setting of the study, from which causal inferences cannot be drawn. A further limitation is that it would be necessary to assess patients during follow-up to ensure longitudinal stability. Although one of the inclusion criteria was that the control group did not have FH, it would be interesting to consider an FH^+^ control group. It has also been assumed that various combinations of FH carry equivalent genetic loadings. However, patients could have different genetic loadings (one vs. multiple family members affected). Another limitation is that type of cannabis use was not measured. Finally, future studies should analyse cognition in relatives of patients, in addition to the relationship between cognition, FH of psychosis, and extent of cannabis use.

Although further studies are required to improve the understanding of the influence of FH on the association between cannabis use and cognition in FEP patients, our findings show that cannabis use is associated with better performance in patients with an FH of psychosis and a worse performance in those with no FH of psychosis in the verbal-memory, executive-function and global-cognition domains.

## Supporting Information

S1 Table Cognitive domain scores depending on having family history and on cannabis use at baseline. (DOC)
