# Supplementary material for: Opposite Cannabis-Cognition Associations in Psychotic Patients Depending on Family History
Source: PLoS One. 2016 Aug 11;11(8):e0160949. doi: 10.1371/journal.pone.0160949 (PMC4981356; doi:10.1371/journal.pone.0160949)
Supplement: S1 Table — (DOC) [file pone.0160949.s001.doc]

**Supplemental Table 1: Cognitive domain scores depending on having family history and on cannabis use at baseline.***

|  | **Controls** | | **Patients** | | | |
| --- | --- | --- | --- | --- | --- | --- |
| **Cognitive domain** | **Non-users (n=200)** | **Cannabis users (n=37)** | **FH^-^ non-users (n=107)** | **FH^-^ cannabis users (n=73)** | **FH^+^ non-users (n=54)** | **FH^+^ cannabis users (n=34)** |
| Processing speed | 0.03 (0.76) | -0.11 (0.96) | -0.96 (1.09) | -0.93 (0.95) | -1.40 (1.11) | -0.91 (0.75) |
| Attention | 0.03 (0.97) | -0.18 (1.17) | 0.34 (1.13) | 0.49 (0.66) | 0.65 (0.63) | 0.27 (0.88) |
| Verbal memory | 0.06 (0.88) | -0.25 (0.86) | -1.08 (1.07) | -1.26 (0.90) | -1.41 (1.02) | -0.69 (1.02) |
| Working memory | 0.62 (0.90) | 0.41 (0.81) | -0.02 (2.55) | 0.11 (0.99) | -0.51 (0.82) | -0.07 (0.88) |
| Executive function | -0.01 (0.35) | 0.02 (0.40) | -0.37 (0.55) | -0.45 (0.45) | -0.46 (0.41) | -0.38 (0.41) |
| Global Cognitive Index | -0.02 (0.41) | -0.12 (0.36) | -0.29 (0.60) | -0.38 (0.31) | -0.52 (0.42) | -0.31 (0.27) |

^*^ Statistical parameters expressed as means (±SD) of the Z-scores.
